# Comorbidity and Disease Activity in Multiple Sclerosis

**DOI:** 10.1001/jamaneurol.2024.2920

**Published:** 2024-09-18

**Authors:** Amber Salter, Samantha Lancia, Kaarina Kowalec, Kathryn C. Fitzgerald, Ruth Ann Marrie

**Affiliations:** 1 Department of Neurology, Section on Statistical Planning and Analysis, UT Southwestern Medical Center, Dallas, Texas; 2 Peter O’Donnell Jr. Brain Institute, University of Texas Southwestern Medical Center, Dallas; 3 College of Pharmacy, Rady Faculty of Health Sciences, University of Manitoba, Winnipeg, Manitoba, Canada; 4 Department of Medical Epidemiology & Biostatistics, Karolinska Institutet, Solna, Sweden; 5 Department of Neurology, Johns Hopkins School of Medicine, Baltimore, Maryland; 6 Department of Internal Medicine, Max Rady College of Medicine, Rady Faculty of Health Sciences, University of Manitoba, Winnipeg, Manitoba, Canada; 7 Department of Community Health Sciences, Max Rady College of Medicine, Rady Faculty of Health Sciences, University of Manitoba, Winnipeg, Manitoba, Canada

## Abstract

**Importance:**

Multiple studies suggest that comorbidity worsens clinically relevant outcomes in multiple sclerosis (MS), including the severity of disability at diagnosis and rate of disability worsening after diagnosis. However, less is known regarding the association of comorbidity with measures of disease activity, such as relapse rate and magnetic resonance imaging lesion accrual, which are relevant to clinicians and clinical trialists.

**Objective:**

To evaluate the association of comorbidities with disease activity in clinical trials of disease-modifying therapies (DMTs) in populations with MS.

**Design, Setting, and Participants:**

A 2-stage meta-analytic approach was used in this cohort study of individual participant data from phase 3 clinical trials of MS DMTs that had 2 years of follow-up and were conducted from November 2001 to March 2018. Data were analyzed from February 2023 to June 2024.

**Exposure:**

Comorbidity burden and individual comorbidities present at trial enrollment, including hypertension; hyperlipidemia; functional cardiovascular disease, ischemic heart, cerebrovascular, and peripheral vascular disease; diabetes; autoimmune thyroid and miscellaneous autoimmune conditions; migraine; lung and skin conditions; depression; anxiety; and other psychiatric disorders.

**Main Outcomes and Measures:**

The main outcome was evidence of disease activity (EDA) over 2 years of follow-up, defined as confirmed relapse activity, disability worsening, or any new lesions on magnetic resonance imaging.

**Results:**

A total of 16 794 participants with MS were included from 17 clinical trials (67.2% female). Over the 2-year follow-up, 61.0% (95% CI, 56.2%-66.3%; *I*^2^ = 97.9%) of the pooled trials had EDA. After adjusting for multiple factors, the presence of 3 or more comorbidities was associated with an increased hazard of EDA (adjusted hazard ratio [AHR], 1.14; 95% CI, 1.02-1.28) compared with no comorbidity. Presence of 2 or more cardiometabolic conditions was also associated with an increased hazard of EDA (AHR, 1.21; 95% CI, 1.08-1.37) compared with no cardiometabolic comorbidity. Presence of 1 psychiatric disorder was associated with an increased hazard of EDA (AHR, 1.07; 95% CI, 1.02-1.14).

**Conclusions and Relevance:**

In this study, a higher burden of comorbidity was associated with worse clinical outcomes in people with MS, although comorbidity could potentially be a partial mediator of other negative prognostic factors. Our findings suggest a substantial adverse association of the comorbidities investigated with MS disease activity and that prevention and management of comorbidities should be a pressing concern in clinical practice.

## Key Points

**Question:**

Are comorbidities associated with disease activity using clinical and imaging outcomes in phase 3 clinical trials of disease-modifying therapies for multiple sclerosis (MS)?

**Findings:**

In this cohort study applying a meta-analytic approach to individual participant clinical trial data including 16 794 persons with MS, a higher burden of comorbidities at trial enrollment was associated with an increased hazard of any disease activity (disability worsening, relapse, or lesion activity) pooled across trials.

**Meaning:**

The adverse association of comorbidities with disease activity suggests that prevention and management of comorbidities in patients with MS should be a pressing clinical concern.

## Teaser

This cohort study uses a meta-analytic approach with data from phase 3 clinical trials of disease-modifying therapies to evaluate the association of comorbidities with disease activity in people with multiple sclerosis.

## Introduction

Multiple sclerosis (MS) demonstrates considerable variability in outcomes. Multiple observational studies have suggested that comorbidity is associated with worse clinically relevant outcomes in MS, including the severity of disability at diagnosis and rate of disability worsening after diagnosis.^1^ Comorbidity may also be associated with lower likelihood of starting a disease-modifying therapy (DMT).^2,3^ However, studies of the association of comorbidity with measures of disease activity, such as relapse rate, are limited, and findings have differed,^4,5^ possibly due to differences in relapse measurement, frequency of assessment, and modest sample sizes.^6,7,8^ These knowledge gaps are relevant to clinicians when prognosticating and making treatment recommendations and to clinical trial design.

Previously, our group investigated the association of comorbidities with clinical outcome measures among participants with relapsing remitting MS in the CombiRx phase 3 clinical trial.^9^ Comorbidity was common in this trial population, with dyslipidemia and anxiety associated with an increased hazard of disease activity, while migraine was associated with a reduced hazard^9^; the latter finding conflicted with prior studies.^4^ The study suggested that well-controlled clinical trial populations may be suitable for evaluating the effects of comorbidities on MS disease processes and that comorbidity burden in trial populations may influence outcomes. Yet, definitive conclusions cannot be drawn from a single clinical trial population. Larger studies with rigorous measures of disease activity and progression are needed. We also found that persons with MS and comorbidity are present in many clinical trials; when pooled across 17 trials, almost half of participants had at least 1 comorbidity.^10^ Therefore, we evaluated the association between comorbidity and disease activity using clinical and imaging outcomes in phase 3 clinical trials of MS DMTs. We hypothesized that a greater overall burden of comorbidities and dyslipidemia and anxiety, in particular, would be associated with shorter time to first relapse and that vascular comorbidities would be associated with faster disability progression and imaging lesion activity.^4,9,11,12,13^

## Methods

### Study Design

We used a 2-stage meta-analytic approach in a cohort study of individual participant data from phase 3 clinical trials of MS DMTs with 2 years of follow-up conducted between November 2001 and March 2018; data were obtained from multiple industry sponsors. Use of the Maelstrom retrospective harmonization guidelines ensured consistency of our approach across studies and allowed pooling across studies.^14^ This study was designated as non–human participant research by the UT Southwestern Medical Center institutional review board. Trial sponsors obtained written informed consent and reviewed data access requests to ensure they were consistent with consents obtained for the trials. We followed the Strengthening the Reporting of Observational Studies in Epidemiology (STROBE) reporting guideline.

### Trials and Populations

Individual participant data were requested from trial sponsors. The trials, populations, and harmonization approach have been described previously.^10^ Subsequently, 2 more trials became available and were approved for inclusion (eTable 1 in Supplement 1).^15^ Data were harmonized using the same approach. The intent-to-treat population from each trial was analyzed (the eMethods in Supplement 1 gives details).

### Comorbidities

Details regarding the classification of comorbidities in the clinical trials are described elsewhere.^10^ In brief, chronic conditions recommended by the International Advisory Committee on Clinical Trials in MS, which included depression, anxiety, hypertension, hyperlipidemia, migraine, diabetes, and chronic lung disease (including asthma and chronic obstructive pulmonary disease) and additional comorbidities, including autoimmune thyroid disease, other psychiatric disorders (eg, bipolar disorder, personality disorder), cerebrovascular disease, functional cardiovascular diseases, ischemic heart disease, peripheral vascular disease (PVD), other miscellaneous autoimmune conditions, and skin conditions, were identified based on medical history data reported at trial enrollment only.^16^ The number of comorbidities (comorbidity burden) was summed (maximum of 15) and categorized as 0, 1, 2, or 3 or more. Cardiometabolic (hypertension, hyperlipidemia, functional cardiovascular, ischemic heart disease, PVD, and diabetes) and psychiatric (depression, anxiety, and other psychiatric disorders) conditions were also summed and categorized as 0, 1, or 2 or more.

### Clinical Outcome

The primary clinical outcome was evidence of disease activity (EDA) over 2 years of follow-up, defined as confirmed disability worsening (measured using the Expanded Disability Status Scale [EDSS]; score ranges from 0 to 10, with higher scores indicating higher levels of disability), relapse activity, or any new or enlarging lesions on magnetic resonance imaging (cumulative unique active lesions).^17^ Secondary outcomes examined each of these components individually (the eMethods in Supplement 1 gives details).

### Covariates

Demographic and clinical characteristics at baseline included sex, age, disability level (measured using the EDSS), treatment assignment (intervention vs placebo or active comparator), disease duration, and number of relapses in the prior year. Age was treated as categorical because several trials reported age in only 5-year increments.^18,19,20,21^ We based disease duration on year of symptom onset when reported and diagnosis year otherwise. Body mass index (BMI) and current smoking status (yes or no) were included in sensitivity analyses when available.

### Statistical Analysis

The association of comorbidity (burden or individual) with clinical outcomes was evaluated using a 2-stage meta-analysis approach. In the first stage, we fitted Cox proportional hazards regression models to evaluate the association of comorbidity burden, each comorbidity, and multiple comorbidities simultaneously with an outcome. We adjusted for age, sex, treatment assignment, disease duration, number of relapses in the prior year, and baseline EDSS. In the second stage, the adjusted hazard ratio (AHR) and 95% CI for each trial were pooled using random-effects meta-analysis with the Der-Simonian and Laird estimator.^22^ The pooled AHR (95% CI) for models are reported. The heterogeneity of findings across studies was assessed using the *I*^2^ statistic, by which values of 0% to 50% indicated low heterogeneity and 51% to 100% indicated high heterogeneity.^23^ Sensitivity analyses included examining the results of trials reporting BMI (continuous) and current smoking status. Additionally, we considered the exploratory outcome of annualized disability change and constructed 99% CIs in consideration of the number of models evaluated (eMethods in Supplement 1). Data were analyzed from February 2023 to June 2024. Analysis was conducted in SAS, version 9.4 (SAS Institute Inc).

## Results

### Trials

After excluding 2 trials^18,24^ that had only 1 year of follow-up in the randomized treatment portion, 17 trials^15,19,20,21,25,26,27,28,29,30,31,32,33,34,35^ including 16 794 individuals (67.2% female; 32.3% male) were analyzed (eTable 2 in Supplement 1). The prevalence of 1 or more comorbidities among trial participants was 45.4%, consistent with previous work by our group^10^ (eTable 3 in Supplement 1). Over the 2-year follow-up, 61.0% (95% CI, 56.2%-66.3%; *I*^2^ = 97.9%) of the pooled trials had EDA. The pooled proportions of each outcome are reported in eTable 4 in Supplement 1.

### Any EDA

**Figure 1.**
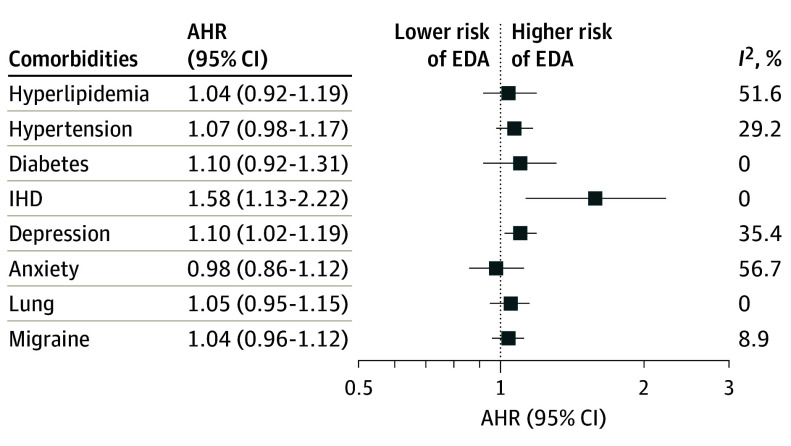
Pooled Adjusted Hazard Ratios (AHRs) for Evidence of Disease Activity (EDA) Models adjusted for included comorbidities, age, sex, treatment assignment, number of relapses in the year before enrollment, baseline Expanded Disability Status Scale, and disease duration. IHD indicates ischemic heart disease.

Presence of 3 or more comorbidities was associated with increased hazard of disease activity after adjusting for age, sex, treatment assignment disease duration, number of relapses in the prior year, and baseline EDSS (AHR, 1.14; 95% CI, 1.02-1.28) compared with no comorbidity, but there was no association for 1 or 2 comorbidities (Table). Presence of 2 or more cardiometabolic conditions was also associated with an increased hazard of disease activity (AHR, 1.21; 95% CI, 1.08-1.37) compared with no cardiometabolic comorbidity. Of these cardiometabolic conditions, ischemic heart disease, hypertension, and cerebrovascular conditions were associated with EDA (Table). Presence of 1 psychiatric disorder was associated with increased hazard of EDA (AHR, 1.07; 95% CI, 1.02-1.14) compared with no psychiatric disorders. Depression was associated with an increased hazard of EDA (AHR, 1.11; 95% CI, 1.03-1.20) compared with no depression. When considering comorbidities jointly, ischemic heart disease and depression were associated with increased hazard of EDA (Figure 1).

**Table.** Pooled Adjusted Hazard Ratios for Clinical Outcomes^a^

Comorbidity	Adjusted hazard ratio (95% CI)			
	Evidence of disease activity	Disability worsening	Relapse	Combined unique active lesions
Total, No.				
0	1 [Reference]	1 [Reference]	1 [Reference]	1 [Reference]
1	1.03 (0.98-1.09)	1.05 (0.96-1.15)	1.10 (1.02-1.18)	0.92 (0.85-1.00)
2	1.07 (0.99-1.16)	1.24 (1.11-1.39)	1.16 (1.04-1.30)	0.91 (0.83-0.99)
≥3	1.14 (1.02-1.28)	1.31 (1.05-1.64)	1.16 (1.01-1.34)	0.89 (0.73-1.07)
Cardiometabolic, No.				
0	1 [Reference]	1 [Reference]	1 [Reference]	1 [Reference]
1	1.03 (0.94-1.12)	0.99 (0.88-1.12)	0.98 (0.89-1.07)	0.94 (0.87-1.02)
≥2	1.21 (1.08-1.37)	1.34 (1.12-1.60)	1.07 (0.90-1.27)	1.06 (0.90-1.24)
Psychiatric, No.				
0	1 [Reference]	1 [Reference]	1 [Reference]	1 [Reference]
1	1.07 (1.02-1.14)	1.18 (1.08-1.29)	1.15 (1.07-1.24)	0.93 (0.87-1.00)
≥2	1.13 (0.94-1.36)	1.39 (1.07-1.79)	1.25 (1.03-1.51)	0.90 (0.75-1.09)
Hyperlipidemia	1.09 (0.96-1.23)	1.16 (1.01-1.34)	1.11 (0.98-1.25)	0.92 (0.82-1.03)
Hypertension	1.09 (1.00-1.18)	1.01 (0.89-1.15)	0.99 (0.90-1.10)	0.99 (0.90-1.09)
Diabetes	1.11 (0.93-1.32)	1.29 (0.98-1.71)	0.95 (0.74-1.21)	1.29 (1.01-1.65)
Ischemic heart disease	1.63 (1.17-2.28)	2.14 (1.21-3.81)	1.70 (0.97-2.96)	2.10 (1.38-3.20)
Functional CVD	0.99 (0.86-1.14)	1.15 (0.90-1.46)	1.03 (0.85-1.24)	0.90 (0.73-1.10)
Cerebrovascular conditions	1.70 (1.12-2.57)	3.20 (1.84-5.56)	1.99 (0.94-4.21)	1.48 (0.85-2.59)
PVD	1.14 (0.84-1.55)	1.71 (1.01-2.90)	1.52 (0.96-2.40)	1.22 (0.84-1.76)
Depression	1.11 (1.03-1.20)	1.29 (1.17-1.43)	1.21 (1.08-1.35)	0.92 (0.83-1.02)
Anxiety	1.02 (0.89-1.16)	1.12 (0.94-1.33)	1.15 (0.98-1.35)	0.88 (0.78-0.99)
Other psychiatric disorder	1.11 (0.96-1.29)	1.13 (0.91-1.40)	1.10 (0.94-1.29)	1.05 (0.87-1.25)
Lung condition	1.08 (0.98-1.18)	1.19 (1.03-1.37)	1.17 (1.03-1.33)	0.89 (0.78-1.03)
Migraine	1.07 (0.98-1.16)	1.13 (0.99-1.29)	1.19 (1.07-1.34)	0.96 (0.86-1.08)
Skin condition	1.05 (0.93-1.18)	1.18 (0.97-1.45)	1.07 (0.91-1.25)	1.04 (0.90-1.21)
Autoimmune thyroid condition	0.95 (0.72-1.26)	1.44 (1.02-2.04)	0.97 (0.72-1.31)	0.79 (0.59-1.06)
Miscellaneous autoimmune condition	1.06 (0.76-1.50)	1.03 (0.54-1.96)	0.92 (0.60-1.40)	1.10 (0.72-1.68)

Abbreviations: CVD, cardiovascular disease; PVD, peripheral vascular disease.

^a^ Models adjusted for age, sex, treatment assignment, number of relapses in the year before enrollment, baseline Expanded Disability Status Scale, and disease duration. Separate models were conducted for each burden of comorbidity and individual comorbidity.

### Disability Worsening

**Figure 2.**
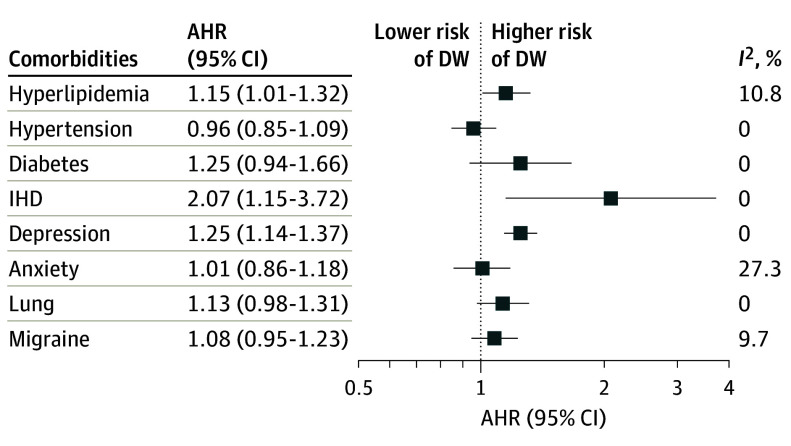
Pooled Adjusted Hazard Ratios (AHRs) for Disability Worsening (DW) Models adjusted for included comorbidities, age, sex, treatment assignment, number of relapses in the year before enrollment, baseline Expanded Disability Status Scale, and disease duration. IHD indicates ischemic heart disease.

Presence of 2 comorbidities was associated with an increased hazard of disability worsening (AHR, 1.24; 95% CI, 1.11-1.39) compared with no comorbidity, as was presence of 3 or more comorbidities (AHR, 1.31; 95% CI, 1.05-1.64) (Table). Presence of 2 or more cardiometabolic conditions was associated with disability worsening (AHR, 1.34; 95% CI, 1.12-1.60), and hyperlipidemia, ischemic heart disease, cerebrovascular conditions, and PVD were associated with an increased hazard of disability worsening (Table). Presence of 1 or 2 or more psychiatric disorders was associated with a greater hazard of disability worsening (Table), and depression was associated with an increased hazard of disability worsening (AHR, 1.29; 95% CI, 1.17-1.43) compared with no depression (Table) and when considered with other comorbidities (Figure 2). Lung conditions were associated with an increased hazard of disability worsening (AHR, 1.19; 95% CI, 1.03-1.37) (Table).

### Relapse

**Figure 3.**
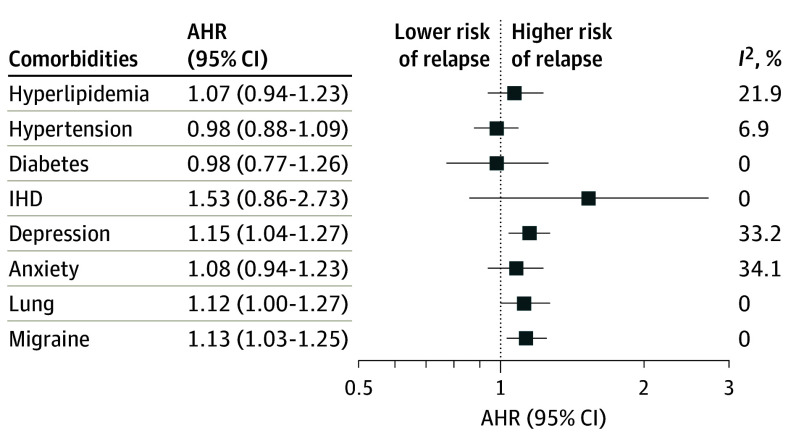
Pooled Adjusted Hazard Ratios (AHRs) for Relapse Models adjusted for included comorbidities, age, sex, treatment assignment, number of relapses in the year before enrollment, baseline Expanded Disability Status Scale, and disease duration. IHD indicates ischemic heart disease.

Compared with no comorbidity, the adjusted hazard of relapse increased with greater comorbidity burden (Table). Cardiometabolic conditions were not associated with relapse activity. Presence of 1 or 2 or more psychiatric disorders was associated with an increased hazard of relapse (1 disorder: AHR, 1.15 [95% CI, 1.07-1.24]; ≥2 disorders: AHR, 1.25 [95% CI, 1.03-1.51]) compared with no psychiatric disorders (Table). Depression was associated with an increased hazard of relapse (AHR, 1.21; 95% CI, 1.08-1.35) compared with no depression (Table) and when considered with other comorbidities (Figure 3). Lung conditions and migraine were associated with an increased hazard of relapse (Table and Figure 3).

### Cumulative Unique Active Lesions

**Figure 4.**
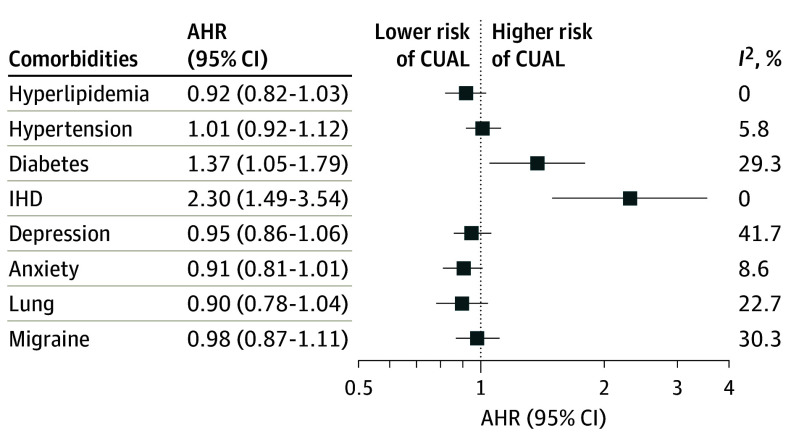
Pooled Adjusted Hazard Ratios (AHRs) for Combined Unique Activity Lesion (CUAL) Models adjusted for included comorbidities, age, sex, treatment assignment, number of relapses in the year before enrollment, baseline Expanded Disability Status Scale, and disease duration. IHD indicates ischemic heart disease.

The pooled proportion with any cumulative unique active lesions was 40.1% (95% CI, 34.6%-46.5%; *I*^2^ = 98.5%), with a pooled proportion of gadolinium-enhancing lesions of 17.0% (95% CI, 14.8%-19.5%; *I*^2^ = 93.3%) and new or enlarging T2 lesions of 39.3% (95% CI, 33.7%-45.8%; *I*^2^ = 98.6%). The number of comorbidities (all, cardiometabolic, and psychiatric) was not associated with an elevated hazard of developing new lesions (Table). An increased hazard of new lesion activity was observed for ischemic heart disease and diabetes after adjusting for other comorbidities and additional covariates (Table and Figure 4).

### Sensitivity Analyses

Results were generally consistent when including BMI (n = 16) (eTables 5-8 in Supplement 1). Presence of 2 comorbidities was associated with increased annualized disability change compared with no comorbidities (eTable 9 in Supplement 1), while cardiometabolic and psychiatric comorbidities combined and individually were not consistently observed to have a greater change in annualized disability. Implementing wider, 99% CIs resulted in a limited number of associations with 99% CIs that did not include 1 when the 95% threshold also did not include 1 (eTables 10-13 in Supplement 1). The results for smoking showed some consistency with the model results; however, with only 6 trials capturing smoking status, the comparability of the results with the main models was limited (eTables 5-8 in Supplement 1).

## Discussion

With the use of a meta-analytic approach, this cohort study investigated the association between comorbidity and disease activity in 17 phase 3 clinical trials of DMTs involving 16 794 individuals with MS. A greater burden of comorbidity was associated with adverse outcomes. Specifically, a greater number of comorbidities, including burden of cardiometabolic and psychiatric comorbidities, were associated with a greater risk of relapse and disability worsening. These findings persisted for cardiometabolic conditions and depression after adjusting for multiple demographic and clinical factors.

The association of a higher burden of comorbidity with worse clinical outcomes in MS is consistent with findings of other observational studies.^4,36,37^ Based on self-reported comorbidity, having 3 or more comorbidities was associated with a 45% increased rate of relapse.^4^ We found an increased risk of relapse among those with comorbidities and nominally higher hazard for 1 and 2 comorbidities after adjusting for BMI. While we adjusted for similar factors as those adjusted for in the study by Kowalec et al,^4^ differences in the number of comorbidities considered, the population and baseline prevalence of comorbidities, and differences in comorbidity measurement (eg, case report forms, self-report, and administrative data) may contribute to differences in the strength of associations. Yet, the presence of these associations across different methods and data sources demonstrated consistency of the findings and strengthens the evidence for these associations. While this study was conducted in clinical trial populations and controlled for DMT use in the analysis, higher comorbidity burden has been associated with lower hazard of initiating DMTs,^3^ and multiple studies have suggested that comorbidity affects DMT adherence and persistence.^2,38,39,40^ The implications for the broader population with MS suggests that the effects of higher comorbidity burden could be greater and have consequences for the clinical care for people with MS beyond the comorbidities.

Cardiometabolic conditions have been associated with worsening of clinical outcome measures, including progression and relapse in other studies.^4,11,12,41,42^ In a cross-sectional analysis of the MS Partners Advancing Technology Health Solutions (MS PATHS) cohort, a higher number of vascular comorbidities was associated with poorer outcomes on performance-based measures of cognition and limb function.^43^ Similarly, we observed an increased rate of any disease activity and disability progression that persisted after adjusting for BMI but a nominally higher rate of relapse among those with 2 or more cardiometabolic conditions. The results when smoking was included were generally consistent, although the 95% CIs were wider likely due to fewer number of trials collecting information on smoking. One 5-year cohort study^38^ reported that a 1-point increase in the Framingham Risk Score was associated with a 31% higher risk of relapse and a 19% increased risk of reaching an EDSS of 6. We also found associations for ischemic heart disease and hyperlipidemia, with clinical outcomes similar to those in other studies.^4,36,43^ Collectively, evidence suggests that the accumulation of these conditions in persons with MS over time may be associated with their disease course. The association, if left unaddressed, may increase in magnitude over time, especially given that the prevalence of vascular comorbidities increases with age and persons with MS have increased prevalence of these conditions compared with the general population.^1,42,44,45^

Two cohort studies using population-based administrative data reported that depression was associated with faster disability progression measured using the EDSS.^13,46^ We also found an association of depression and burden of psychiatric disorder with disability worsening and relapse. Previously, our group identified an association with relapse and anxiety in the CombiRx trial^10^; however, not all trials examined in the present study showed an association after fully adjusting for multiple factors. This may reflect differences in prevalence of comorbidity across trials, as in the present study, the pooled prevalence of anxiety was lower compared with that in the CombiRx trial alone (8% vs 12%, respectively) and some heterogeneity among trials was observed.

Knowledge of the association of chronic lung conditions and migraine with clinical outcomes is limited. A survey study in England did not identify an association of asthma with risk of reaching EDSS scores of 4 and 6 after controlling for confounders.^47^ Yet, a US-based study found an association with obstructive lung disease and self-reported disability.^27^ While we observed some differences among models, chronic lung conditions were associated with disability progression and relapse. Increasing incidence of these conditions worldwide and the elevated prevalence in persons with MS indicate that more investigation regarding the impact of treating these conditions in patients with MS is warranted.^48,49^ Migraine was associated with a 13% increased hazard of relapse after adjusting for many factors and in the presence of other comorbidities. The estimate decreased within in the bounds of the effect estimate identified in a Canadian 2-year prospective multicenter cohort study (adjusted rate ratio, 1.38; 95% CI, 1.01-1.89).^4^ Although a case-control study found that individuals with MS and migraine had more contrast-enhancing lesions compared with control individuals without migraine,^50^ we did not. Studies of imaging outcomes have primarily focused on individual comorbidities.^51^ In our study, lesion activity was not associated with comorbidity burden. Given studies reporting an association of atrophy-related outcomes with disability,^51^ future studies to explore the association of comorbidity with atrophy-related outcomes appear to be warranted.

Our findings have important implications. Clinically, cardiovascular, mental health, and chronic lung conditions are the most common comorbidities that affect people with MS throughout their disease course.^51^ While mental health conditions have a consistently high prevalence at all ages, the prevalence of cardiovascular and chronic lung conditions increases with age.^48^ Thus, the substantial adverse associations of these comorbidities with relapse and disability progression suggest that the prevention and management of comorbidities should be a pressing clinical concern. Identification of optimal care models and teams for managing comorbidity in patients with MS is still needed,^51^ as are clinical trials that establish whether treating comorbidity improves outcomes and whether or not management of comorbidity for people with MS should differ from that in the general population. Our findings also raise the question as to whether efficacy of DMT differs in people with comorbidities. From a clinical trial perspective, we found that people with MS and comorbidities participated in trials and that their burden of comorbidity had an adverse association with event rates, which may have consequences that affect future trial sample sizes and duration.

### Strengths and Limitations

Our study summarized the association of comorbidity burden and individual comorbidities across a large number of phase 3 clinical trials of MS DMTs. These rigorously collected data included well-adjudicated MS outcomes with improved accuracy compared with assessment in clinical practice (eg, disability worsening using the EDSS). The data were harmonized carefully using a protocol based on established guidelines.

The study also has limitations. Comorbidity status was determined using medical history data, the recording of which likely varied across trials. Standardized questionnaires to query explicitly rather than volunteer comorbidity would improve future efforts to examine comorbidities.^16^ The data also lacked information on severity and timing of the comorbidity. If only less severe comorbidities were included, this may have led to underestimation of the association between comorbidity and clinical outcomes. Additionally, investigation of the role of severity and treatment of comorbidity should be the subject of future studies. The inability to understand the temporality of the comorbidity and health behaviors limited our ability to determine whether our findings could reflect comorbidity acting as a partial mediator of the association of obesity or smoking with clinical outcomes. By design, we used an approach to potentially limit reverse causality, ensuring comorbidity was measured before clinical outcomes. The use of meta-analysis identified robust associations, limiting the possibility of bias or chance accounting for the findings. However, unmeasured confounding could still occur. As our focus was on estimating the association between comorbidity and clinical outcomes, we used a reductionist approach that considered EDA as a composite clinical outcome. It is possible that some spurious associations were observed with the findings. Despite the presence of individuals with comorbidities in these clinical trials, clinical trial populations are not necessarily representative of the general population with MS and may lack diversity.^52^ The prevalence of many of the comorbidities considered varies by characteristics, such as race, ethnicity, and age, and this could have implications for future trial designs. Finally, some comorbidities are rare, and clinical event rates were fairly low, which together reduced our ability to detect associations.

## Conclusions

With the use of a meta-analytic approach, this cohort study investigated the association of comorbidity with MS outcomes in phase 3 clinical trials of DMTs. The study found that a higher burden of comorbidity was associated with an increased hazard of EDA and specifically higher relapse rates and faster disability progression in people with MS participating in the trials. As these findings are coherent with observational studies^4,11,12,36,37,41,42,43^ using different study designs and data sources, evidence suggests that clinicians need to routinely address comorbidity in people with MS.

## Notes

Supplement 1.
eMethods.
**eTable 1**. Clinical trials available for inclusion (*italicized* were excluded) **eTable 2**. Demographic and clinical characteristics of trial participants **eTable 3.** Pooled prevalence of comorbidities among the included trials **eTable 4.** Pooled proportion of clinical outcomes **eTable 5.** Associations of comorbidity with evidence of disease activity in MS clinical trial populations **eTable 6.** Associations of comorbidity with disability worsening in MS clinical trial populations **eTable 7.** Associations of comorbidity with relapse in MS clinical trial populations **eTable 8.** Associations of comorbidity with lesion activity in MS clinical trial populations **eTable 9.** Associations of comorbidity with annualized disability change in MS clinical trial populations **eTable 10.** Pooled adjusted hazard ratios and 99% confidence intervals for evidence of disease activity by comorbidity status **eTable 11.** Pooled adjusted hazard ratios and 99% confidence intervals for disability worsening by comorbidity status **eTable 12.** Pooled adjusted hazard ratios and 99% confidence intervals for relapse by comorbidity status **eTable 13.** Pooled adjusted hazard ratios and 99% confidence intervals for combined unique active lesions by comorbidity status

Supplement 2.
Data Sharing Statement
