# Supplementary material for: Comorbidity and Disease Activity in Multiple Sclerosis
Source: JAMA Neurol. 2024 Sep 18;81(11):1170–7. doi: 10.1001/jamaneurol.2024.2920 (PMC11411448; doi:10.1001/jamaneurol.2024.2920)
Supplement: Supplement 1. — eMethods. eTable 1. Clinical trials available for inclusion (italicized were excluded) eTable 2. Demographic and clinical characteristics of trial participants eTable 3. Pooled prevalence of comorbidities among the included trials eTable 4. Pooled proportion of clinical outcomes eTable 5. Associations of comorbidity with evidence of disease activity in MS clinical trial populations eTable 6. Associations of comorbidity with disability worsening in MS clinical trial populations eTable 7. Associations of comorbidity with relapse in MS clinical trial populations eTable 8. Associations of comorbidity with lesion activity in MS clinical trial populations eTable 9. Associations of comorbidity with annualized disability change in MS clinical trial populations eTable 10. Pooled adjusted hazard ratios and 99% confidence intervals for evidence of disease activity by comorbidity status eTable 11. Pooled adjusted hazard ratios and 99% confidence intervals for disability worsening by comorbidity status eTable 12. Pooled adjusted hazard ratios and 99% confidence intervals for relapse by comorbidity status eTable 13. Pooled adjusted hazard ratios and 99% confidence intervals for combined unique active lesions by comorbidity status [file jamaneurol-e242920-s001.pdf]

Supplemental Online Content

Salter A, Lancia S, Kowalec K, Fitzgerald KC, Marrie RA. Comorbidity and disease activity in multiple sclerosis. *JAMA Neurol.* Published online September 18, 2024. doi:10.1001/jamaneurol.2024.2920

eMethods.

eTable 1. Clinical trials available for inclusion (*italicized* were excluded)

eTable 2. Demographic and clinical characteristics of trial participants

eTable 3. Pooled prevalence of comorbidities among the included trials

eTable 4. Pooled proportion of clinical outcomes

eTable 5. Associations of comorbidity with evidence of disease activity in MS clinical trial populations

eTable 6. Associations of comorbidity with disability worsening in MS clinical trial populations

eTable 7. Associations of comorbidity with relapse in MS clinical trial populations

eTable 8. Associations of comorbidity with lesion activity in MS clinical trial populations

eTable 9. Associations of comorbidity with annualized disability change in MS clinical trial populations

eTable 10. Pooled adjusted hazard ratios and 99% confidence intervals for evidence of disease activity by comorbidity status

eTable 11. Pooled adjusted hazard ratios and 99% confidence intervals for disability worsening by comorbidity status

eTable 12. Pooled adjusted hazard ratios and 99% confidence intervals for relapse by comorbidity status

eTable 13. Pooled adjusted hazard ratios and 99% confidence intervals for combined unique active lesions by comorbidity status

This supplemental material has been provided by the authors to give readers additional information about their work.

© 2024 Salter A et al. *JAMA Neurology.*

eMethods

Trials and Populations:

We excluded trials if the study duration was <2 years.[18,24] One trial had a placebo and active comparator arm and only the placebo arm was included.[25] This trial only performed imaging in a subset of participants and we utilized the full sample for non-imaging clinical outcomes.[25]

Analysis.

For trials with extensive missing covariate data, we included the models without the covariate(s). INFORMS was missing >50% for disease duration (n missing=392 [67.8%]) and ORATORIO did not capture the number of relapses 1-year prior.

Sensitivity analyses included examining the results for the trials that captured BMI (continuous), and current smoking status. BMI was included in 16 trials and current smoking status was captured in 6 trials. We considered the exploratory outcome of annualized disability change (eMethods). Additionally, 99% confidence intervals were constructed in consideration of the number of models evaluated.

Disease Activity Outcomes:

Confirmed disability worsening was defined as a 1-point increase in the Expanded Disability Status Scale (EDSS) if the baseline EDSS was ≤5.0 and 0.5-point increase in the EDSS if the baseline EDSS was ≥5.5, sustained for 6 months. For CombiRx[26], we used protocol and non-protocol defined relapses to define a relapse to improve comparability with other trials.[9] Imaging outcomes summarized the number of new gadolinium enhancing lesions, new non-enhancing T2 lesions, and enlarged non-enhancing T2 lesions observed post-enrollment.

Annualized EDSS change was calculated as the change in EDSS each year over two years of follow-up. This outcome was analyzed using a repeated measures linear model using an interaction term between time and comorbidity to evaluate differences in the annualized rate of change.

© 2024 Salter A et al. *JAMA Neurology.*

eTable 1. Clinical trials available for inclusion.

Study acronym	Clinicaltrials.gov number	Year	Indication	Test substance	Comparator	Sample size, n	Age criteria, y	EDSS criteria
AFFIRM	NCT00027300	2006	RRMS	Natalizumab	Placebo	942	18–50	0–5.0
SENTINEL	NCT00030966	2006	RRMS	Natalizumab	Interferon β-1a	1,171	18–55	0–5.0
COMBIRX	NCT00211887	2006	RRMS	Interferon β-1a and Glatiramer Acetate	Interferon β-1a, Glatiramer Acetate	1,008	18–60	0–5.5
FREEDOMS	NCT00289978	2006	RRMS	Fingolimod	Placebo	1,272	18–55	0–5.5
FREEDOMS II	NCT00355134	2006	RRMS	Fingolimod	Placebo	1,083	18–55	0–5.5
CLARITY	NCT00213135	2010	RRMS	Cladribine	Placebo	1,326	18–65	0–5.5
TRANSFORMS	*NCT00340834*	*2010*	*RRMS*	*Fingolimod*	*Interferon β-1a*	*1,292*	*18–55*	*0–5.0*
CARE-MS I	NCT00530348	2012	RRMS	Alemtuzumab	Interferon β-1a	563	18–50	0–3.0
CARE-MS II	NCT00548405	2012	RRMS	Alemtuzumab	Interferon β-1a	798	18–55	0–5.0
CONFIRM	NCT00451451	2012	RRMS	Dimethyl fumarate	Placebo, Glatiramer Acetate	1,417	18–55	0–5.0
DEFINE	NCT00420212	2012	RRMS	Dimethyl fumarate	Placebo	1,234	18–55	0–5.0
ADVANCE	*NCT00906399*	*2014*	*RRMS*	*PEG-interferon β-1a*	*Placebo*	*1,512*	*18–65*	*0–5.0*
INFORMS	NCT00731692	2016	PPMS	Fingolimod	Placebo	823	25–65	3.5–6.0
OPERA I	NCT01247324	2016	RMS	Ocrelizumab	Interferon β-1a	821	18–55	0–5.5
OPERA II	NCT01412333	2016	RMS	Ocrelizumab	Interferon β-1a	835	18–55	0–5.5
ORATORIO	NCT01194570	2016	PPMS	Ocrelizumab	Placebo	732	18–55	3.0–6.5
ASCEND	NCT01416181	2018	SPMS	Natalizumab	Placebo	887	18–58	3.0–6.5
ASCLEPIOS I	NCT02792218	2020	RMS	Ofatumumab	Teriflunomide	927	18–55	0–5.5
ASCLEPIOS II	NCT02792231	2020	RMS	Ofatumumab	Teriflunomide	955	18–55	0–5.5

RRMS = relapsing remitting multiple sclerosis, RMS= relapsing multiple sclerosis, PPMS= primary progressive multiple sclerosis, SPMS= secondary progressive multiple sclerosis, EDSS= Expanded Disability Status Scale.
Studies italicized were excluded because the study duration was less than 2years.

© 2024 Salter A et al. *JAMA Neurology.*

eTable 2. Demographic and clinical characteristics of trial participants

**Baseline**	**AFFIRM (N=942)**	**SENTINEL (N=1196)**	**COMBIRX (N=1008)**	**FREEDOMS (N=1272)**	**FREEDOMS II (N=1083)**	**CLARITY (N=1327)**	**CARE-MS I (N=581)**	**CARE-MS II (N=798)**
**Sex**								
Female	660(70.1)	875(73.2)	730(72.4)	889(69.9)	844(77.9)	899(67.7)	376(64.7)	562(66.9)
Male	282(29.9)	321(26.8)	278(27.6)	383(30.1)	239(22.1)	428(32.3)	205(35.3)	278(33.1)
**Age categories**								
>18 - 30	268(28.5)	180(15.1)	261(25.9)	319(26.1)	149(13.8)	282(21.4)	247(43.9)	255(32.0)
>30 - 40	352(37.4)	483(40.4)	351(34.8)	453(37.0)	349(32.2)	433(32.8)	199(35.3)	325(40.7)
>40 - 50	322(34.2)	475(39.7)	278(27.6)	369(30.2)	438(40.4)	416(31.5)	115(20.4)	189(23.7)
>50+	0 (0.0)	58(4.8)	118(11.7)	82(6.7)	147(13.6)	188(14.3)	2(0.36)	29(3.6)
**Race**								
White	899(95.4)	1117(93.4)	888(88.1)	1213(95.4)	962(88.8)	1618(98.1)	549(94.5)	752(89.5)
Other	43(4.6)	79(6.6)	120(11.9)	59(4.6)	121(11.2)	32(1.9)	32(5.5)	88(10.5)
**Total Num. of Comorbidity**								
0	613(65.1)	481(40.2)	380(37.7)	764(60.1)	246(22.7)	984(74.2)	383(68.0)	383(48.0)
1	223(23.7)	378(31.6)	329(32.6)	345(27.1)	297(27.4)	252(19.0)	98(17.4)	194(24.3)
2	81(8.6)	209(17.5)	186(18.5)	121(9.5)	279(25.8)	66(5.0)	48(8.5)	119(14.9)
3+	25(2.7)	128(10.7)	113(11.2)	42(3.3)	261(24.1)	25(1.9)	34(6.0)	102(12.8)
**EDSS,** median [25th, 75th]	2.0 [1.5, 3.0]	2.5 [1.5, 3.0]	2.0 [1.0, 2.5]	2.0 [1.5, 3.5]	2.5 [1.5, 3.5]	3.0 [2.0, 4.0]	2.0 [1.5, 2.5]	2.5 [2.0, 3.5]
**Num. of Relapses past year**	1.0 [1.0, 2.0]	1.0 [1.0, 2.0]	2.0 [1.0, 2.0]	1.0 [1.0, 2.0]	1.0 [1.0, 2.0]	1.0 [0.0, 1.0]	2.0 [1.0,2.0]	1.0 [1.0, 2.0]
**Disease duration,** mean (SD)	7.5(6.6)	9.2(6.5)	4.3(5.7)	8.2(6.6)	10.6(8.0)	8.7(7.4)	2.1(1.3)	4.5(2.7)
**Body Mass Index,** mean (SD)		25.7(5.7)	28.7(6.8)	24.6(4.6)	27.6(6.1)	24.9(5.7)	25.0(4.9)	26.4(6.0)
**Current Smoker**	---	---	220(26.2)	357(28.1)	261(24.1)	---	---	---

© 2024 Salter A et al. *JAMA Neurology.*

Baseline	CONFIRM (N=1429)	DEFINE (N=1237)	OPERA I (N=821)	OPERA II (N=835)	ALCEPIOS I (N=927)	ALCEPIOS II (N = 955)	INFORMS (N=970)	ORATORIO (N=732)	ASCEND (N=889)
**Sex**									
Female	1002(70.1)	911(73.6)	542(66.0)	551(66.0)	635(65.5)	638(66.3)	469(48.4)	361(49.3)	550(61.9)
Male	427(29.9)	326(26.4)	279(34.0)	284(34.0)	292(31.5)	162(33.7)	501(51.6)	371(50.7)	339(38.1)
**Age categories**									
>18 - 30	382(26.7)	260(21.0)	225(27.4)	212(25.4)	170(18.5)	211(22.1)	12(1.2)	40(5.5)	29(3.3)
>30 - 40	493(34.5)	434(35.1)	299(36.4)	324(38.8)	322(5.0)	377(39.5)	145(14.9)	171(23.4)	139(15.6)
>40 - 50	431(30.2)	435(35.2)	228(27.8)	231(27.7)	316(34.3)	179(18.7)	368(37.9)	316(43.2)	373(42.0)
>50+	123(8.6)	108(8.7)	69(8.4)	68(8.1)	112(12.2)	188(19.7)	445(45.9)	205(28.0)	348(39.1)
**Race**									
White	1202(84.1)	971(78.5)	750(91.4)	750(89.8)	102(11.0)	---	---	689(94.1)	231(26.0)
Other	227(15.9)	266(21.5)	71(8.6)	85(10.2)	823(89.0)	---	---	43(5.9)	11(1.2)
**Total Num. of Comorbidity**									
0	891(62.4)	695(56.2)	485(59.1)	477(57.1)	415 (44.8)	447(46.8)	441(45.5)	395(54.0)	521(58.6)
1	324(22.7)	302(24.4)	214(26.1)	214(25.6)	261(28.2)	232(24.3)	305(31.4)	191(26.1)	211(23.7)
2	130(9.1)	154(12.4)	87(10.6)	89(10.7)	110 (11.9)	113(11.8)	160(16.5)	88(12.0)	106(11.9)
3+	84(5.9)	86(7.0)	35(4.3)	55(6.6)	141(15.2)	163(17.1)	64(6.6)	58(7.9)	51(5.7)
**EDSS,** median [25th, 75th]	2.5 [1.5, 3.5]	2.0 [1.5, 3.5]	2.5 [2.0, 3.5]	2.5 [2.0, 3.5]	3.0 [2.0, 4.0]	3.0 [2.0, 4.0]	4.5 [4.0, 5.5]	4.5 [3.5, 6.0]	6.0 [5.0, 6.5]
**Num. of Relapses past year**	1.0 [1.0, 2.0]	1.0 [1.0, 2.0]	1.0 [1.0, 2.0]	1.0 [1.0, 1.0]	1.0 [1.0, 2.0]	1.0 [1.0, 2.0]	---	---	0.0 [0.0, 0.0]
**Disease duration,** mean (SD)	7.7(6.3)	8.3(6.6)	6.5(6.2)	6.7(6.1)	8.3(7.0)	8.2(7.4)	5.8(2.5)	6.5(3.9)	16.4(7.4
**Body Mass Index,** mean (SD)	25.4(5.8)	25.3(5.7)	26.1(6.0)	26.4(6.0)	26.2(6.3)	25.5(5.9)	25.4(4.3)	24.9(4.9)	25.3(5.3)
**Current Smoker**	---	---	---	---	87(9.4)	91(9.5)	236(34.4)	---	---

© 2024 Salter A et al. *JAMA Neurology*.

eTable 3. Pooled prevalence of comorbidities among the included trials.

Comorbidity	Level	Pooled Prevalence (95%CI)
Total Number of Comorbidities	0	54.6 (48.1, 61.0)
	1	25.7 (23.6, 27.9)
	2	11.8 (9.69, 14.2)
	3+	6.4 (4.58, 8.69)
Cardiometabolic Comorbidities	0	76.7 (71.5, 81.3)
	1	17.9 (14.6, 21.6)
	2+	4.9 (3.49, 6.67)
Psychiatric Comorbidities	0	82.4 (77.5, 86.5)
	1	14.0 (11.0, 17.6)
	2+	3.4 (2.25, 4.90)
Hyperlipidemia		7.6 (5.43, 10.3)
Hypertension		10.6 (8.79, 12.6)
Diabetes		1.4 (0.88, 2.22)
Ischemic heart disease		0.4 (0.26, 0.60)
Functional CVD		2.2 (1.69, 2.94)
Cerebrovascular conditions		0.3 (0.22, 0.48)
PVD		0.6 (0.47, 0.75)
Depression		17.5 (13.8, 21.8)
Anxiety		7.9 (5.93, 10.3)
Other Psychiatric		3.0 (2.43, 3.79)
Lung condition		4.9 (3.69, 6.35)
Migraine		8.5 (6.33, 11.2)
Skin condition		0.7 (0.44, 1.22)
Autoimmune thyroid condition		1.2 (0.94, 1.54)
Miscellaneous autoimmune condition		0.5 (0.37, 0.74)

CVD, Cardiovascular disease; PVD, Peripheral vascular disease

© 2024 Salter A et al. *JAMA Neurology.*

eTable 4. Pooled proportion of clinical outcomes

**Clinical Outcome**	**Event Proportion (95%CI)**	**I^2**
Evidence of Disease Activity	61.0% (56.2%, 66.3%)	97.9
Disability Worsening	17.7% (15.3%, 20.5%)	95.5
Relapse	25.0% (21.3%, 29.3%)	97.9
Combined Unique Active Lesions	40.1% (34.6%, 46.5%)	98.5
Gadolinium-enhancing lesions	17.0% (14.8%, 19.5%)	93.3
New or enlarging T2 lesions	39.3% (33.7%, 45.8%)	98.6

© 2024 Salter A et al. *JAMA Neurology*.

eTable 5. Associations of comorbidity with evidence of disease activity in MS clinical trial populations

**Evidence of Disease Activity[#]**					
**Pooled Adjusted Hazard Ratios (95%CI)**					
**Comorbidity**	**Level**	**Model 1***	**Model 2****	**Model 2+ BMI**	**Model 2+ Smoking (n=6)**
Total Number of Comorbidities	0	Reference	Reference	Reference	Reference
	1	1.05 (0.99, 1.11)	1.03 (0.98, 1.09)	1.01 (0.95, 1.07)	0.98 (0.90, 1.07)
	2	1.08 (0.99, 1.18)	1.07 (0.99, 1.16)	1.05 (0.97, 1.13)	0.96 (0.85, 1.08)
	3+	**1.17 (1.04, 1.32)**	**1.14 (1.02, 1.28)**	**1.13 (1.00, 1.27)**	1.03 (0.87, 1.23)
Cardiometabolic	0	Reference	Reference	Reference	Reference
	1	1.02 (0.94, 1.11)	1.03 (0.94, 1.12)	1.01 (0.92, 1.11)	1.02 (0.92, 1.12)
	2+	**1.19 (1.06, 1.34)**	**1.21 (1.08, 1.37)**	**1.20 (1.06, 1.36)**	1.16 (0.98, 1.38)
Psychiatric	0	Reference	Reference	Reference	Reference
	1	**1.09 (1.03, 1.15)**	**1.07 (1.02, 1.14)**	**1.07 (1.00, 1.13)**	0.99 (0.88, 1.13)
	2+	1.13 (0.95, 1.36)	1.13 (0.94, 1.36)	1.12 (0.94, 1.32)	1.09 (0.97, 1.22)
Hyperlipidemia		1.09 (0.98, 1.22)	1.09 (0.96, 1.23)	1.07 (0.96, 1.19)	1.10 (0.80, 1.51)
Hypertension		1.08 (0.99, 1.18)	**1.09 (1.00, 1.18)**	1.07 (0.98, 1.16)	**1.80 (1.18, 2.76)**
Diabetes		1.09 (0.91, 1.29)	1.11 (0.93, 1.32)	1.08 (0.89, 1.30)	0.92 (0.75, 1.14)
Ischemic heart disease		**1.59 (1.14, 2.21)**	**1.63 (1.17, 2.28)**	**1.62 (1.16, 2.28)**	1.42 (0.78, 2.57)
Functional CVD		1.01 (0.88, 1.16)	0.99 (0.86, 1.14)	1.00 (0.86, 1.15)	1.33 (0.89, 2.00)
Cerebrovascular conditions		**1.76 (1.16, 2.69)**	**1.70 (1.12, 2.57)**	**1.77 (1.16, 2.70)**	1.02 (0.91, 1.14)
PVD		1.08 (0.80, 1.46)	1.14 (0.84, 1.55)	1.16 (0.84, 1.59)	0.98 (0.81, 1.20)
Depression		**1.12 (1.03, 1.23)**	**1.11 (1.03, 1.20)**	**1.06 (1.00, 1.13)**	1.06 (0.88, 1.28)
Anxiety		1.03 (0.90, 1.17)	1.02 (0.89, 1.16)	1.02 (0.89, 1.18)	1.00 (0.87, 1.14)
Other Psychiatric		1.14 (0.98, 1.32)	1.11 (0.96, 1.29)	**1.16 (1.02, 1.33)**	1.02 (0.89, 1.17)
Lung condition		**1.09 (1.00, 1.20)**	1.08 (0.98, 1.18)	1.05 (0.96, 1.16)	1.00 (0.79, 1.28)
Migraine		1.09 (0.99, 1.19)	1.07 (0.98, 1.16)	1.06 (0.97, 1.15)	0.82 (0.55, 1.24)
Skin condition		1.03 (0.91, 1.16)	1.05 (0.93, 1.18)	1.04 (0.92, 1.17)	0.73 (0.42, 1.25)
Autoimmune thyroid condition		0.98 (0.74, 1.28)	0.95 (0.72, 1.26)	0.90 (0.67, 1.22)	0.99 (0.88, 1.13)
Miscellaneous autoimmune condition		1.16 (0.76, 1.76)	1.06 (0.76, 1.50)	1.00 (0.69, 1.43)	1.09 (0.97, 1.22)

[#]Separate models were conducted for each burden of comorbidity and individual comorbidity.

*Models adjusted for age, sex and treatment assignment.

**Models adjusted for age, sex, treatment assignment, baseline EDSS, disease duration and number of relapses in the year prior to enrollment.

CVD, Cardiovascular disease; PVD, Peripheral vascular disease

© 2024 Salter A et al. *JAMA Neurology.*

eTable 6. Associations of comorbidity with disability worsening in MS clinical trial populations

**Disability Worsening#**					
**Pooled Adjusted Hazard Ratios (95%CI)**					
**Comorbidity**	**Level**	**Model 1***	**Model 2****	**Model 2+ BMI**	**Model 2+ Smoking (n=6)**
Total Number of Comorbidities	0	Reference	Reference	Reference	Reference
	1	1.03 (0.94, 1.12)	1.05 (0.96, 1.15)	1.01 (0.92, 1.11)	0.90 (0.78, 1.03)
	2	**1.18 (1.05, 1.33)**	**1.24 (1.11, 1.39)**	**1.19 (1.05, 1.34)**	1.08 (0.87, 1.34)
	3+	1.20 (0.95, 1.51)	**1.31 (1.05, 1.64)**	**1.35 (1.06, 1.73)**	1.21 (0.91, 1.62)
Cardiometabolic	0	Reference	Reference	Reference	Reference
	1	1.00 (0.90, 1.10)	0.99 (0.88, 1.12)	0.97 (0.85, 1.11)	1.04 (0.88, 1.23)
	2+	**1.24 (1.05, 1.47)**	**1.34 (1.12, 1.60)**	**1.31 (1.09, 1.58)**	**1.47 (1.13, 1.89)**
Psychiatric	0	Reference	Reference	Reference	Reference
	1	**1.10 (1.01, 1.20)**	**1.18 (1.08, 1.29)**	**1.17 (1.06, 1.29)**	1.17 (0.98, 1.39)
	2+	1.20 (0.92, 1.57)	**1.39 (1.07, 1.79)**	**1.47 (1.15, 1.88)**	1.03 (0.68, 1.57)
Hyperlipidemia		1.09 (0.98, 1.22)	**1.16 (1.01, 1.34)**	**1.13 (1.00, 1.29)**	1.14 (0.96, 1.36)
Hypertension		1.08 (0.99, 1.18)	1.01 (0.89, 1.15)	1.00 (0.88, 1.15)	1.13 (0.95, 1.35)
Diabetes		1.09 (0.91, 1.29)	1.29 (0.98, 1.71)	1.18 (0.87, 1.59)	1.42 (0.75, 2.67)
Ischemic heart disease		**1.59 (1.14, 2.21)**	**2.14 (1.21, 3.81)**	**2.04 (1.17, 3.55)**	1.78 (0.88, 3.60)
Functional CVD		1.01 (0.88, 1.16)	1.15 (0.90, 1.46)	1.10 (0.86, 1.41)	0.98 (0.70, 1.37)
Cerebrovascular conditions		**1.76 (1.16, 2.69)**	**3.20 (1.84, 5.56)**	**3.21 (1.84, 5.58)**	**3.22 (1.59, 6.52)**
PVD		1.08 (0.80, 1.46)	**1.71 (1.01, 2.90)**	1.35 (0.74, 2.46)	1.51 (0.67, 3.40)
Depression		**1.12 (1.03, 1.23)**	**1.29 (1.17, 1.43)**	**1.27 (1.14, 1.41)**	1.20 (0.97, 1.49)
Anxiety		1.03 (0.90, 1.17)	1.12 (0.94, 1.33)	**1.17 (1.01, 1.36)**	1.02 (0.82, 1.28)
Other Psychiatric		1.14 (0.98, 1.32)	1.13 (0.91, 1.40)	1.19 (0.95, 1.49)	1.07 (0.78, 1.46)
Lung condition		**1.09 (1.00, 1.20)**	**1.19 (1.03, 1.37)**	1.14 (0.98, 1.33)	1.12 (0.87, 1.44)
Migraine		1.09 (0.99, 1.19)	1.13 (0.99, 1.29)	1.07 (0.94, 1.21)	1.05 (0.85, 1.30)
Skin condition		1.03 (0.91, 1.16)	1.18 (0.97, 1.45)	1.12 (0.89, 1.40)	1.18 (0.79, 1.75)
Autoimmune thyroid condition		0.98 (0.74, 1.28)	**1.44 (1.02, 2.04)**	1.41 (0.99, 1.99)	1.47 (0.73, 2.97)
Miscellaneous autoimmune condition		1.16 (0.76, 1.76)	1.03 (0.54, 1.96)	0.95 (0.40, 2.23)	1.35 (0.46, 3.97)

#Separate models were conducted for each burden of comorbidity and individual comorbidity.

*Models adjusted for age, sex and treatment assignment.

**Models adjusted for age, sex, treatment assignment, baseline EDSS, disease duration and number of relapses in the year prior to enrollment.

CVD, Cardiovascular disease; PVD, Peripheral vascular disease

© 2024 Salter A et al. *JAMA Neurology.*

eTable 7. Associations of comorbidity with relapse in MS clinical trial populations

Relapse[#]					
**Pooled Adjusted Hazard Ratios (95%CI)**					
**Comorbidity**	**Level**	**Model 1***	**Model 2****	**Model 2+ BMI**	**Model 2+ Smoking (n=6)**
Total Number of Comorbidities	0	Reference	Reference	Reference	Reference
	1	**1.11 (1.03, 1.21)**	**1.10 (1.02, 1.18)**	1.06 (0.98, 1.16)	1.02 (0.85, 1.23)
	2	**1.17 (1.06, 1.30)**	**1.16 (1.04, 1.30)**	1.09 (0.97, 1.22)	1.03 (0.88, 1.22)
	3+	**1.24 (1.06, 1.46)**	**1.16 (1.01, 1.34)**	**1.18 (1.01, 1.38)**	1.11 (0.84, 1.48)
Cardiometabolic	0	Reference	Reference	Reference	Reference
	1	0.99 (0.90, 1.09)	0.98 (0.89, 1.07)	0.94 (0.85, 1.04)	0.99 (0.85, 1.14)
	2+	1.09 (0.93, 1.29)	1.07 (0.90, 1.27)	1.08 (0.90, 1.29)	1.09 (0.82, 1.43)
Psychiatric	0	Reference	Reference	Reference	Reference
	1	**1.20 (1.11, 1.31)**	**1.15 (1.07, 1.24)**	**1.14 (1.04, 1.24)**	**1.22 (1.01, 1.47)**
	2+	**1.27 (1.05, 1.55)**	**1.25 (1.03, 1.51)**	**1.31 (1.05, 1.62)**	1.15 (0.80, 1.65)
Hyperlipidemia		1.09 (0.98, 1.22)	1.11 (0.98, 1.25)	1.07 (0.95, 1.21)	1.06 (0.84, 1.33)
Hypertension		1.03 (0.91, 1.16)	0.99 (0.90, 1.10)	0.98 (0.87, 1.09)	0.99 (0.83, 1.17)
Diabetes		1.02 (0.80, 1.29)	0.95 (0.74, 1.21)	0.98 (0.75, 1.27)	1.10 (0.72, 1.67)
Ischemic heart disease		**1.85 (1.06, 3.21)**	1.70 (0.97, 2.96)	1.70 (0.99, 2.92)	1.40 (0.69, 2.86)
Functional CVD		1.07 (0.89, 1.28)	1.03 (0.85, 1.24)	1.00 (0.81, 1.23)	0.83 (0.60, 1.15)
Cerebrovascular conditions		1.86 (0.88, 3.93)	1.99 (0.94, 4.21)	1.97 (0.93, 4.18)	1.10 (0.27, 4.45)
PVD		1.44 (0.92, 2.24)	1.52 (0.96, 2.40)	1.40 (0.84, 2.35)	1.83 (0.85, 3.92)
Depression		**1.24 (1.12, 1.39)**	**1.21 (1.08, 1.35)**	**1.19 (1.05, 1.34)**	1.15 (0.90, 1.46)
Anxiety		1.16 (0.99, 1.36)	1.15 (0.98, 1.35)	1.17 (0.98, 1.41)	1.13 (0.86, 1.48)
Other Psychiatric		**1.20 (1.02, 1.39)**	1.10 (0.94, 1.29)	1.16 (0.97, 1.38)	1.27 (0.98, 1.63)
Lung condition		**1.20 (1.07, 1.35)**	**1.17 (1.03, 1.33)**	**1.15 (1.00, 1.31)**	1.01 (0.80, 1.27)
Migraine		**1.19 (1.05, 1.35)**	**1.19 (1.07, 1.34)**	**1.18 (1.04, 1.35)**	1.16 (0.96, 1.39)
Skin condition		1.09 (0.93, 1.26)	1.07 (0.91, 1.25)	1.01 (0.86, 1.20)	1.05 (0.83, 1.34)
Autoimmune thyroid condition		1.01 (0.76, 1.35)	0.97 (0.72, 1.31)	0.92 (0.66, 1.28)	0.82 (0.42, 1.61)
Miscellaneous autoimmune condition		1.04 (0.71, 1.52)	0.92 (0.60, 1.40)	0.94 (0.57, 1.55)	0.78 (0.31, 1.93)

[#]Separate models were conducted for each individual and burden of comorbidity.

*Models adjusted for age, sex and treatment assignment.

**Models adjusted for age, sex, treatment assignment, baseline EDSS, disease duration and number of relapses in the year prior to enrollment.

CVD, Cardiovascular disease; PVD, Peripheral vascular disease

© 2024 Salter A et al. *JAMA Neurology.*

eTable 8. Associations of comorbidity with lesion activity in MS clinical trial populations

**Cumulative Unique Active Lesions#**					
**Pooled Adjusted Hazard Ratios (95%CI)**					
**Comorbidity**	**Level**	**Model 1***	**Model 2****	**Model 2+ BMI**	**Model 2+ Smoking (n=6)**
Total Number of Comorbidities	0	Reference	Reference	Reference	Reference
	1	0.93 (0.86, 1.02)	0.92 (0.85, 1.00)	0.91 (0.84, 1.00)	0.98 (0.82, 1.17)
	2	0.93 (0.85, 1.01)	0.91 (0.83, 0.99)	0.92 (0.83, 1.01)	0.89 (0.75, 1.05)
	3+	0.90 (0.73, 1.10)	0.89 (0.73, 1.07)	0.87 (0.73, 1.05)	0.79 (0.58, 1.10)
Cardiometabolic	0	Reference	Reference	Reference	Reference
	1	0.92 (0.84, 1.01)	0.94 (0.87, 1.02)	0.94 (0.86, 1.03)	0.98 (0.84, 1.14)
	2+	1.05 (0.90, 1.23)	1.06 (0.90, 1.24)	1.07 (0.90, 1.26)	0.96 (0.72, 1.28)
Psychiatric	0	Reference	Reference	Reference	Reference
	1	0.95 (0.89, 1.02)	0.93 (0.87, 1.00)	0.93 (0.86, 1.00)	0.96 (0.82, 1.11)
	2+	0.92 (0.76, 1.11)	0.90 (0.75, 1.09)	0.88 (0.73, 1.06)	**0.74 (0.56, 0.98)**
Hyperlipidemia		0.91 (0.82, 1.02)	0.92 (0.82, 1.03)	0.92 (0.82, 1.03)	0.91 (0.79, 1.05)
Hypertension		0.99 (0.90, 1.10)	0.99 (0.90, 1.09)	0.98 (0.88, 1.09)	0.98 (0.81, 1.19)
Diabetes		**1.28 (1.03, 1.59)**	**1.29 (1.01, 1.65)**	**1.32 (1.02, 1.70)**	1.16 (0.79, 1.69)
Ischemic heart disease		**2.13 (1.41, 3.23)**	**2.10 (1.38, 3.20)**	**2.02 (1.32, 3.11)**	**2.43 (1.44, 4.09)**
Functional CVD		0.90 (0.75, 1.08)	0.90 (0.73, 1.10)	0.92 (0.74, 1.15)	0.91 (0.64, 1.30)
Cerebrovascular conditions		1.45 (0.83, 2.53)	1.48 (0.85, 2.59)	1.32 (0.73, 2.36)	1.62 (0.75, 3.49)
PVD		1.23 (0.85, 1.77)	1.22 (0.84, 1.76)	1.36 (0.93, 2.00)	1.38 (0.87, 2.21)
Depression		0.94 (0.85, 1.05)	0.92 (0.83, 1.02)	0.90 (0.81, 0.99)	0.94 (0.80, 1.10)
Anxiety		**0.89 (0.79, 0.99)**	**0.88 (0.78, 0.99)**	**0.87 (0.77, 0.97)**	0.78 (0.64, 0.95)
Other Psychiatric		1.06 (0.88, 1.27)	1.05 (0.87, 1.25)	1.09 (0.90, 1.33)	0.86 (0.68, 1.09)
Lung condition		0.91 (0.80, 1.03)	0.89 (0.78, 1.03)	0.91 (0.78, 1.06)	0.97 (0.76, 1.25)
Migraine		0.95 (0.84, 1.07)	0.96 (0.86, 1.08)	1.00 (0.90, 1.12)	0.89 (0.75, 1.07)
Skin condition		1.06 (0.90, 1.24)	1.04 (0.90, 1.21)	1.04 (0.89, 1.21)	1.08 (0.80, 1.47)
Autoimmune thyroid condition		0.77 (0.58, 1.03)	0.79 (0.59, 1.06)	0.79 (0.59, 1.07)	0.66 (0.42, 1.05)
Miscellaneous autoimmune condition		1.05 (0.75, 1.46)	1.10 (0.72, 1.68)	1.21 (0.69, 2.10)	1.37 (0.73, 2.56)

#Separate models were conducted for each individual and burden of comorbidity.

*Models adjusted for age, sex and treatment assignment.

**Models adjusted for age, sex, treatment assignment, baseline EDSS, disease duration and number of relapses in the year prior to enrollment.

CVD, Cardiovascular disease; PVD, Peripheral vascular disease

© 2024 Salter A et al. *JAMA Neurology.*

eTable 9. Associations of comorbidity with annualized disability change in MS clinical trial populations

**Annualized Disability Change#**					
**Pooled Adjusted Hazard Ratios (95%CI)**					
**Comorbidity**	**Level**	**Model 1***	**Model 2****	**Model 2+ BMI**	**Model 2+ Smoking (n=6)**
Total Number of Comorbidities	0	Reference	Reference	Reference	Reference
	1	0.03 (-0.07, 0.13)	0.01 (-0.09, 0.10)	0.004 (-0.10, 0.10)	0.01 (-0.13, 0.10)
	2	**0.11 (0.02, 0.20)**	**0.10 (0.01, 0.20)**	**0.11 (0.01, 0.20)**	**0.15 (0.01, 0.27)**
	3+	0.04 (-0.09, 0.16)	0.05 (-0.09, 0.18)	0.08 (-0.08, 0.24)	0.03 (-0.20, 0.26)
Cardiometabolic	0	Reference	Reference	Reference	Reference
	1	0.05 (-0.05, 0.14)	0.05 (-0.04, 0.15)	0.06 (-0.04, 0.17)	0.08 (-0.08, 0.24)
	2+	0.17 (-0.11, 0.44)	0.16 (-0.12, 0.44)	0.07 (-0.17, 0.30)	0.09 (-0.13, 0.31)
Psychiatric	0	Reference	Reference	Reference	Reference
	1	0.02 (-0.06, 0.11)	0.02 (-0.07, 0.10)	-0.002 (-0.09, 0.09)	-0.04 (-0.16, 0.07)
	2+	0.04 (-0.14, 0.23)	0.07 (-0.13, 0.26)	0.08 (-0.11, 0.28)	0.02 (-0.23, 0.28)
Hyperlipidemia		0.01 (-0.09, 0.11)	0.01 (-0.10, 0.11)	0.01 (-0.10, 0.12)	0.08 (-0.12, 0.28)
Hypertension		0.06 (-0.04, 0.16)	0.05 (-0.04, 0.15)	0.06 (-0.03, 0.15)	0.07 (-0.09, 0.23)
Diabetes		-0.02 (-0.26, 0.23)	-0.04 (-0.27, 0.19)	-0.04 (-0.25, 0.17)	**0.14 (0.05, 0.23)**
Ischemic heart disease		-1.04 (-3.38, 1.29)	-0.97 (-3.33, 1.39)	-1.10 (-3.33, 1.12)	-0.86 (-3.39, 0.44)
Functional CVD		-0.11 (-0.60, 0.37)	-0.07 (-0.60, 0.45)	0.01 (-0.56, 0.59)	0.20 (-0.05, 0.45)
Cerebrovascular conditions		-1.08 (-2.21, 0.05)	-1.04 (-2.12, 0.04)	-1.15 (-2.31, 0.01)	-1.32 (-3.09, 0.44)
PVD		-0.01 (-0.36, 0.34)	-0.04 (-0.37, 0.30)	0.03 (-0.32, 0.37)	-0.14 (-0.67, 0.38)
Depression		0.05 (-0.04, 0.13)	0.04 (-0.04, 0.13)	0.04 (-0.05, 0.13)	0.03 (-0.09, 0.14)
Anxiety		0.04 (-0.11, 0.19)	0.07 (-0.09, 0.23)	0.07 (-0.10, 0.23)	-0.03 (-0.31, 0.25)
Other Psychiatric		0.03 (-0.13, 0.20)	0.02 (-0.15, 0.19)	0.01 (-0.31, 0.33)	-0.13 (-0.42, 0.17)
Lung condition		0.004 (-0.13, 0.13)	0.02 (-0.12, 0.16)	0.02 (-0.12, 0.16)	-0.01 (-0.19, 0.18)
Migraine		0.09 (-0.07, 0.26)	0.09 (-0.07, 0.24)	**0.14 (0.00, 0.27)**	0.13 (-0.10, 0.37)
Skin condition		0.01 (-0.14, 0.16)	0.03 (-0.13, 0.20)	0.06 (-0.14, 0.25)	-0.03 (-0.19, 0.13)
Autoimmune thyroid condition		0.35 (-0.10, 0.80)	0.37 (-0.10, 0.83)	0.41 (-0.09, 0.90)	0.42 (-0.19, 1.02)
Miscellaneous autoimmune condition		-0.71 (-2.08, 0.65)	-0.62 (-1.94, 0.71)	-0.80 (-2.27, 0.66)	0.24 (-0.62, 1.10)

#Separate models were conducted for each burden of comorbidity and individual comorbidity.

*Models adjusted for age, sex, and treatment assignment.

**Models adjusted for age, sex, treatment assignment, baseline EDSS, disease duration and number of relapses in the year prior to enrollment.

CVD, Cardiovascular disease; PVD, Peripheral vascular disease

© 2024 Salter A et al. *JAMA Neurology*.

eTable 10. Pooled adjusted* hazard ratios and 99% confidence intervals for evidence of disease activity by comorbidity status#.

Evidence of Disease Activity#					
**Pooled Adjusted Hazard Ratios (99%CI)**					
**Comorbidity**	**Level**	**Model 1***	**Model 2****	**Model 2 +BMI**	**Model 2 + Smoking (n=6)**
Total Number of Comorbidities	0	Reference	Reference	Reference	Reference
	1	1.05 (0.97, 1.13)	1.03 (0.95, 1.11)	1.01 (0.93, 1.09)	0.98 (0.86, 1.10)
	2	1.08 (0.95, 1.21)	1.07 (0.95, 1.19)	1.05 (0.94, 1.16)	0.96 (0.80, 1.12)
	3+	1.17 (0.97, 1.37)	1.14 (0.96, 1.32)	1.13 (0.95, 1.31)	1.03 (0.77, 1.29)
Cardiometabolic	0	Reference	Reference	Reference	Reference
	1	1.02 (0.90, 1.14)	1.03 (0.91, 1.15)	1.01 (0.88, 1.14)	1.02 (0.89, 1.15)
	2+	1.19 (0.99, 1.39)	1.21 (1.00, 1.42)	1.20 (0.99, 1.41)	1.16 (0.87, 1.45)
Psychiatric	0	Reference	Reference	Reference	Reference
	1	1.09 (1.01, 1.17)	1.07 (0.98, 1.16)	1.07 (0.99, 1.15)	1.07 (0.95, 1.19)
	2+	1.13 (0.83, 1.43)	1.13 (0.83, 1.43)	1.12 (0.86, 1.38)	0.92 (0.51, 1.33)
Hyperlipidemia		1.09 (0.92, 1.26)	1.09 (0.91, 1.27)	1.07 (0.91, 1.23)	0.99 (0.81, 1.17)
Hypertension		1.08 (0.95, 1.21)	1.09 (0.97, 1.21)	1.07 (0.95, 1.19)	1.09 (0.92, 1.26)
Diabetes		1.09 (0.83, 1.35)	1.11 (0.83, 1.39)	1.08 (0.79, 1.37)	1.10 (0.56, 1.64)
Ischemic heart disease		1.59 (0.78, 2.4)	1.63 (0.78, 2.48)	1.62 (0.75, 2.49)	1.80 (0.54, 3.06)
Functional CVD		1.01 (0.81, 1.21)	0.99 (0.79, 1.19)	1.00 (0.80, 1.2)	0.92 (0.63, 1.21)
Cerebrovascular conditions		1.76 (0.54, 2.98)	1.70 (0.56, 2.84)	1.77 (0.55, 2.99)	1.42 (0.00, 2.93)
PVD		1.08 (0.58, 1.58)	1.14 (0.60, 1.68)	1.16 (0.59, 1.73)	1.33 (0.45, 2.21)
Depression		1.12 (0.98, 1.26)	1.11 (0.99, 1.23)	1.06 (0.97, 1.15)	1.02 (0.86, 1.18)
Anxiety		1.03 (0.85, 1.21)	1.02 (0.84, 1.20)	1.02 (0.81, 1.23)	0.98 (0.69, 1.27)
Other Psychiatric		1.14 (0.9, 1.38)	1.11 (0.87, 1.35)	1.16 (0.94, 1.38)	1.06 (0.77, 1.35)
Lung condition		1.09 (0.95, 1.23)	1.08 (0.95, 1.21)	1.05 (0.91, 1.19)	1.00 (0.82, 1.18)
Migraine		1.09 (0.96, 1.22)	1.07 (0.95, 1.19)	1.06 (0.94, 1.18)	1.02 (0.82, 1.22)
Skin condition		1.03 (0.86, 1.2)	1.05 (0.88, 1.22)	1.04 (0.87, 1.21)	1.00 (0.63, 1.37)
Autoimmune thyroid condition		0.98 (0.59, 1.37)	0.95 (0.54, 1.36)	0.90 (0.48, 1.32)	0.82 (0.27, 1.37)
Miscellaneous autoimmune condition		1.16 (0.37, 1.95)	1.06 (0.48, 1.64)	1.00 (0.43, 1.57)	0.73 (0.05, 1.41)

#Separate models were conducted for each burden of comorbidity and individual comorbidity.

*Models adjusted for age, sex, and treatment assignment.

**Models adjusted for age, sex, treatment assignment, baseline EDSS, disease duration and number of relapses in the year prior to enrollment.

CVD, Cardiovascular disease; PVD, Peripheral vascular disease

© 2024 Salter A et al. *JAMA Neurology*.

eTable 11. Pooled adjusted* hazard ratios and 99% confidence intervals for disability worsening by comorbidity status#.

**Disability Worsening#**					
**Pooled Adjusted Hazard Ratios (99%CI)**					
**Comorbidity**	**Level**	**Model 1***	**Model 2****	**Model 2 +BMI**	**Model 2 + Smoking (n=6)**
Total Number of Comorbidities	0	Reference	Reference	Reference	Reference
	1	1.03 (0.91, 1.15)	1.05 (0.92, 1.18)	1.01 (0.88, 1.14)	0.90 (0.73, 1.07)
	2	1.18 (0.98, 1.38)	1.24 (1.04, 1.44)	1.19 (0.99, 1.39)	1.08 (0.74, 1.42)
	3+	1.20 (0.79, 1.61)	1.31 (0.88, 1.74)	1.35 (0.85, 1.85)	1.21 (0.67, 1.75)
Cardiometabolic	0	Reference	Reference	Reference	Reference
	1	1.00 (0.87, 1.13)	0.99 (0.82, 1.16)	0.97 (0.79, 1.15)	1.04 (0.79, 1.29)
	2+	1.24 (0.94, 1.54)	1.34 (1.00, 1.68)	1.30 (0.96, 1.66)	1.47 (0.92, 2.02)
Psychiatric	0	Reference	Reference	Reference	Reference
	1	1.10 (0.97, 1.23)	1.18 (1.04, 1.32)	1.17 (1.01, 1.33)	1.17 (0.88, 1.46)
	2+	1.20 (0.71, 1.69)	1.39 (0.86, 1.92)	1.45 (0.93, 2.01)	1.03 (0.32, 1.74)
Hyperlipidemia		1.17 (0.95, 1.39)	1.16 (0.92, 1.40)	1.13 (0.92, 1.34)	1.14 (0.85, 1.43)
Hypertension		0.96 (0.80, 1.12)	1.01 (0.83, 1.19)	1.00 (0.80, 1.20)	1.13 (0.84, 1.42)
Diabetes		1.10 (0.65, 1.55)	1.29 (0.74, 1.84)	1.18 (0.64, 1.72)	1.42 (0.00, 3.06)
Ischemic heart disease		1.84 (0.07, 3.61)	2.14 (0.00, 4.33)	2.04 (0.06, 4.02)	1.78 (0.00, 4.17)
Functional CVD		1.06 (0.72, 1.40)	1.15 (0.74, 1.56)	1.10 (0.69, 1.51)	0.98 (0.47, 1.49)
Cerebrovascular conditions		2.92 (0.00, 6.09)	3.20 (0.10, 6.30)	3.21 (0.10, 6.32)	3.22 (0.00, 7.56)
PVD		1.76 (0.21, 3.31)	1.71 (0.15, 3.27)	1.35 (0.00, 2.81)	1.51 (0.00, 3.99)
Depression		1.20 (0.98, 1.42)	1.29 (1.11, 1.47)	1.27 (1.09, 1.45)	1.20 (0.82, 1.58)
Anxiety		1.08 (0.79, 1.37)	1.12 (0.84, 1.40)	1.17 (0.92, 1.42)	1.02 (0.68, 1.36)
Other Psychiatric		1.08 (0.71, 1.45)	1.13 (0.78, 1.48)	1.19 (0.80, 1.58)	1.07 (0.56, 1.58)
Lung condition		1.17 (0.93, 1.41)	1.19 (0.95, 1.43)	1.14 (0.89, 1.39)	1.12 (0.70, 1.54)
Migraine		1.16 (0.94, 1.38)	1.13 (0.92, 1.34)	1.07 (0.89, 1.25)	1.05 (0.72, 1.38)
Skin condition		1.12 (0.80, 1.44)	1.18 (0.83, 1.53)	1.12 (0.75, 1.49)	1.18 (0.43, 1.93)
Autoimmune thyroid condition		1.32 (0.47, 2.17)	1.44 (0.65, 2.23)	1.41 (0.65, 2.17)	1.47 (0.00, 3.44)
Miscellaneous autoimmune condition		1.58 (0.00, 3.28)	1.03 (0.00, 2.25)	0.95 (0.00, 2.63)	1.35 (0.00, 4.79)

#Separate models were conducted for each burden of comorbidity and individual comorbidity.

*Models adjusted for age, sex, and treatment assignment.

**Models adjusted for age, sex, treatment assignment, baseline EDSS, disease duration and number of relapses in the year prior to enrollment.

CVD, Cardiovascular disease; PVD, Peripheral vascular disease

© 2024 Salter A et al. *JAMA Neurology*.

© 2024 Salter A et al. *JAMA Neurology*.

eTable 12. Pooled adjusted* hazard ratios and 99% confidence intervals for relapse by comorbidity status#.

Relapse#					
**Pooled Adjusted Hazard Ratios (99%CI)**					
**Comorbidity**	**Level**	**Model 1***	**Model 2****	**Model 2 +BMI**	**Model 2 + Smoking (n=6)**
Total Number of Comorbidities	0	Reference	Reference	Reference	Reference
	1	1.11 (0.98, 1.24)	1.10 (0.99, 1.21)	1.06 (0.93, 1.19)	1.02 (0.74, 1.30)
	2	1.17 (1.00, 1.34)	1.16 (0.98, 1.34)	1.09 (0.92, 1.26)	1.03 (0.78, 1.28)
	3+	1.24 (0.95, 1.53)	1.16 (0.92, 1.40)	1.18 (0.92, 1.44)	1.11 (0.62, 1.60)
Cardiometabolic	0	Reference	Reference	Reference	Reference
	1	0.99 (0.86, 1.12)	0.98 (0.86, 1.1)	0.94 (0.81, 1.07)	0.99 (0.79, 1.19)
	2+	1.09 (0.83, 1.35)	1.07 (0.81, 1.33)	1.08 (0.80, 1.36)	1.09 (0.64, 1.54)
Psychiatric	0	Reference	Reference	Reference	Reference
	1	1.20 (1.06, 1.34)	1.15 (1.03, 1.27)	1.14 (1.01, 1.27)	1.22 (0.89, 1.55)
	2+	1.27 (0.9, 1.64)	1.25 (0.91, 1.59)	1.31 (0.90, 1.72)	1.15 (0.49, 1.8`1)
Hyperlipidemia		1.09 (0.92, 1.26)	1.11 (0.93, 1.29)	1.07 (0.89, 1.25)	1.06 (0.71, 1.41)
Hypertension		1.03 (0.86, 1.20)	0.99 (0.85, 1.13)	0.98 (0.84, 1.12)	0.99 (0.75, 1.23)
Diabetes		1.02 (0.67, 1.37)	0.95 (0.61, 1.29)	0.98 (0.60, 1.36)	1.10 (0.35, 1.85)
Ischemic heart disease		1.85 (0.06, 3.64)	1.70 (0.04, 3.36)	1.70 (0.10, 3.30)	1.40 (0.00, 3.32)
Functional CVD		1.07 (0.79, 1.35)	1.03 (0.75, 1.31)	1.00 (0.70, 1.30)	0.83 (0.41, 1.25)
Cerebrovascular conditions		1.86 (0.00, 4.58)	1.99 (0.00, 4.91)	1.97 (0.00, 4.87)	1.10 (0.00, 5.50)
PVD		1.44 (0.39, 2.49)	1.52 (0.36, 2.68)	1.40 (0.15, 2.65)	1.83 (0.00, 4.58)
Depression		1.24 (1.04, 1.44)	1.21 (1.03, 1.39)	1.19 (0.99, 1.39)	1.15 (0.74, 1.56)
Anxiety		1.16 (0.90, 1.42)	1.15 (0.89, 1.41)	1.17 (0.85, 1.49)	1.13 (0.67, 1.59)
Other Psychiatric		1.20 (0.95, 1.45)	1.10 (0.85, 1.35)	1.16 (0.87, 1.45)	1.27 (0.80, 1.74)
Lung condition		1.20 (1.00, 1.40)	1.17 (0.96, 1.38)	1.15 (0.94, 1.36)	1.01 (0.67, 1.35)
Migraine		1.19 (0.98, 1.40)	1.19 (0.99, 1.39)	1.18 (0.96, 1.40)	1.16 (0.86, 1.46)
Skin condition		1.09 (0.87, 1.31)	1.07 (0.83, 1.31)	1.01 (0.76, 1.26)	1.05 (0.67, 1.43)
Autoimmune thyroid condition		1.01 (0.56, 1.46)	0.97 (0.52, 1.42)	0.92 (0.45, 1.39)	0.82 (0.00, 1.86)
Miscellaneous autoimmune condition		1.04 (0.41, 1.67)	0.92 (0.29, 1.55)	0.94 (0.14, 1.74)	0.78 (0.00, 2.29)

#Separate models were conducted for each burden of comorbidity and individual comorbidity.

*Models adjusted for age, sex, and treatment assignment.

**Models adjusted for age, sex, treatment assignment, baseline EDSS, disease duration and number of relapses in the year prior to enrollment.

CVD, Cardiovascular disease; PVD, Peripheral vascular disease

© 2024 Salter A et al. *JAMA Neurology.*

eTable 13. Pooled adjusted* hazard ratios and 99% confidence intervals for combined unique active lesions by comorbidity status#.

Cumulative Unique Active Lesions#					
**Pooled Adjusted Hazard Ratios (99%CI)**					
**Comorbidity**	**Level**	**Model 1***	**Model 2****	**Model 2 +BMI**	**Model 2 + Smoking (n=6)**
Total Number of Comorbidities	0	Reference	Reference	Reference	Reference
	1	0.93 (0.81, 1.05)	0.92 (0.81, 1.03)	0.91 (0.79, 1.03)	0.98 (0.73, 1.23)
	2	0.93 (0.82, 1.04)	0.91 (0.79, 1.03)	0.92 (0.80, 1.04)	0.89 (0.68, 1.10)
	3+	0.90 (0.64, 1.16)	0.89 (0.65, 1.13)	0.87 (0.63, 1.11)	0.79 (0.38, 1.20)
Cardiometabolic	0	Reference	Reference	Reference	Reference
	1	0.92 (0.80, 1.04)	0.94 (0.83, 1.05)	0.94 (0.82, 1.06)	0.98 (0.77, 1.19)
	2+	1.05 (0.81, 1.29)	1.06 (0.82, 1.30)	1.07 (0.82, 1.32)	0.96 (0.54, 1.38)
Psychiatric	0	Reference	Reference	Reference	Reference
	1	0.95 (0.86, 1.04)	0.93 (0.84, 1.02)	0.93 (0.84, 1.02)	0.96 (0.76, 1.16)
	2+	0.92 (0.67, 1.17)	0.90 (0.65, 1.15)	0.88 (0.64, 1.12)	0.74 (0.42, 1.06)
Hyperlipidemia		0.91 (0.77, 1.05)	0.92 (0.78, 1.06)	0.92 (0.78, 1.06)	0.91 (0.73, 1.09)
Hypertension		0.99 (0.85, 1.13)	0.99 (0.86, 1.12)	0.98 (0.84, 1.12)	0.98 (0.7, 1.26)
Diabetes		1.28 (0.87, 1.69)	1.29 (0.82, 1.76)	1.32 (0.82, 1.82)	1.16 (0.46, 1.86)
Ischemic heart disease		2.13 (0.68, 3.58)	2.10 (0.65, 3.55)	2.02 (0.59, 3.45)	2.43 (0.25, 4.61)
Functional CVD		0.90 (0.66, 1.14)	0.90 (0.64, 1.16)	0.92 (0.62, 1.22)	0.91 (0.4, 1.42)
Cerebrovascular conditions		1.45 (0.03, 2.87)	1.48 (0.02, 2.94)	1.32 (0.00, 2.69)	1.62 (0.00, 4.08)
PVD		1.23 (0.52, 1.94)	1.22 (0.51, 1.93)	1.36 (0.52, 2.20)	1.38 (0.29, 2.47)
Depression		0.94 (0.80, 1.08)	0.92 (0.79, 1.05)	0.90 (0.78, 1.02)	0.94 (0.73, 1.15)
Anxiety		0.89 (0.76, 1.02)	0.88 (0.74, 1.02)	0.87 (0.74, 1.00)	0.78 (0.56, 1.00)
Other Psychiatric		1.06 (0.78, 1.34)	1.05 (0.79, 1.31)	1.09 (0.77, 1.41)	0.86 (0.56, 1.16)
Lung condition		0.91 (0.75, 1.07)	0.89 (0.71, 1.07)	0.91 (0.71, 1.11)	0.97 (0.60, 1.34)
Migraine		0.95 (0.79, 1.11)	0.96 (0.80, 1.12)	1.00 (0.84, 1.16)	0.89 (0.65, 1.13)
Skin condition		1.06 (0.82, 1.30)	1.04 (0.82, 1.26)	1.04 (0.82, 1.26)	1.08 (0.57, 1.59)
Autoimmune thyroid condition		0.77 (0.43, 1.11)	0.79 (0.44, 1.14)	0.79 (0.42, 1.16)	0.66 (0.15, 1.17)
Miscellaneous autoimmune condition		1.05 (0.51, 1.59)	1.10 (0.34, 1.86)	1.21 (0.04, 2.38)	1.37 (0.00, 2.93)

#Separate models were conducted for each burden of comorbidity and individual comorbidity.

*Models adjusted for age, sex, and treatment assignment.

**Models adjusted for age, sex, treatment assignment, baseline EDSS, disease duration and number of relapses in the year prior to enrollment.

CVD, Cardiovascular disease; PVD, Peripheral vascular disease

© 2024 Salter A et al. *JAMA Neurology.*
