# Supplementary material for: Comorbidity and Disease Activity in Multiple Sclerosis
Source: JAMA Neurol. 2024 Sep 18;81(11):1170–7. doi: 10.1001/jamaneurol.2024.2920 (PMC11411448; doi:10.1001/jamaneurol.2024.2920)
Supplement: Supplement 2. — Data Sharing Statement [file jamaneurol-e242920-s002.pdf]

Data Sharing Statement

Salter. Comorbidity and Disease Activity in Multiple Sclerosis. *JAMA Neurol*. Published September 18, 2024. doi:10.1001/jamaneurol.2024.2920

Data
Data available: No

Additional Information
Explanation for why data not available: The use of the data is governed by data use agreements with the individual sponsors and the corresponding author is not allowed per these agreements to provide individual patient data. Investigators may request access to anonymized individual patient data and redacted trial documents from each sponsor based on their individual processes. Before use of the data, proposals must be approved by the sponsor or an independent review panel, as appropriate, and a signed data sharing agreement obtained. The corresponding author will provide the analysis code for replication of results, upon request.
